# End-user research in support of long-acting systemic antiretroviral delivery systems: insights from qualitative research with providers and target users in South Africa

**DOI:** 10.1186/s12879-022-07907-0

**Published:** 2022-12-08

**Authors:** Morgan S. Brown, Homaira Hanif, Kristen M. Little, Meredith R. Clark, Andrea R. Thurman, Lola Flomen, Gustavo F. Doncel

**Affiliations:** 1 Evidence Department, Population Services International (PSI), 1120 19th Street NW, Suite 600, Washington, DC 20020 USA; 2 CONRAD, Eastern Virginia Medical School, Norfolk, VA USA

**Keywords:** HIV, PrEP implant, South Africa

## Abstract

**Background:**

While oral pre-exposure prophylaxis (PrEP) has been shown to reduce the risk of HIV, challenges such as adhering to a daily-dosing regimen and persistence have emerged as barriers for at-risks populations in South Africa. This qualitative research sought to investigate perceptions of and preferences for a long-acting, biodegradable implantable PrEP product designed to address these barriers.

**Methods:**

To identify and understand motivators, barriers, and preferences for the PrEP implant, we conducted qualitative in-depth interviews (IDIs) among health care providers (HCPs) and target end-users (young women, adolescent girls, and female sex workers) in urban and rural/peri-urban regions of Gauteng Province, South Africa. The IDIs focused on defining values, beliefs, habits, lifestyles, influencers, and information channels for potential PrEP implant end-users.

**Results:**

We conducted 36 IDIs across health care providers and target end-user respondent segments. Respondents had generally positive reactions to the PrEP implant. Most end-users felt that some undesirable aspects of the implant (e.g., side effects, pain during insertion, potential scarring, and inability to remove implant) would be offset by having a highly effective, and long-lasting HIV prevention product. Although some HCPs believed the implantable PrEP would lead to increases in promiscuity and risky sexual behavior, most HCPs saw value in the PrEP implant’s long duration of protection, its biodegradability, and the likelihood of higher adherence relative to oral PrEP.

**Conclusions:**

This study is a first step toward further research needed to demonstrate the demand for a biodegradable, long-acting implantable PrEP and suggests such a product would be accepted by end-users and HCPs in South Africa. This study indicates the need to develop more convenient, discreet, long-acting, and highly effective biomedical HIV prevention options for at-risk populations.

## Background

With an adult prevalence of 20.4% [1], South Africa has the highest number of people living with HIV globally. Though substantial progress against the 90–90–90 goals has been made and incidence rates are declining, the number of new infections each year remains concerningly high. This is especially true for young women, who are more than three times as likely to acquire HIV compared to males of the same age [2]. While the scale-up of HIV prevention services and antiretroviral (ARV) treatment programs in South Africa have substantially reduced AIDS-related deaths over the past decade, the impact of these interventions has been comparatively less successful in preventing new cases of HIV. Further, incident rate reductions have occurred unevenly, with men disproportionally benefitting from efforts to decrease transmission such as the widespread availability of medical male circumcision programs and the relatively higher rates of HIV treatment coverage among women [3]. New HIV prevention options that meet the specific needs of women and girls are urgently required to address this prevention gap.

Consistent use of daily oral pre-exposure prophylaxis (PrEP) has been shown to reduce the risk of HIV from sex by up to 99% [4], and thus represents a promising option to mitigate the risk of HIV acquisition for women and girls. South Africa was the first country in sub-Saharan Africa to roll-out oral PrEP, and to date an estimated 160,000–165,000 people are currently using PrEP in the country [5]. The World Health Organization (WHO) currently recommends PrEP use for HIV-negative individuals at highest risk of infection, such as female sex workers (FSWs), adolescent girls and young women (AGYW), men who have sex with men (MSM), people who inject drugs, and those with partners who are living with HIV or who have an unknown HIV status [6]. Despite these recommendations and the potential effectiveness of oral PrEP, uptake and adherence remain very low [7]. While oral PrEP is highly effective when taken consistently [8], effectiveness diminishes substantially when individuals are partially or insufficiently adherent [4], particularly among females. Unfortunately, studies suggest that young women struggle to achieve the levels of oral PrEP adherence required for HIV protection [9, 10]. Current adherence and adoption barriers in South Africa are myriad, including: a lack of motivation to take a daily pill, stigma, and frequent visits to a health care provider for monitoring and regular testing [11].

New biomedical HIV prevention options, including long-acting injectables, vaginal rings, and—more recently—implantable PrEP, will likely address some of the current oral PrEP barriers [12]. Products such as the PrEP implant explored in this study are not user-mediated and therefore would not require users to take a daily pill or make frequent clinic visits for prescription refills, making the product potentially more discreet. In South Africa, analogous products such as the contraceptive implant are accepted and in demand amongst women and girls [13–15]. However, little is known about the acceptability of an implantable PrEP product amongst potential end-users and healthcare providers. This qualitative research aimed to inform the development and future scale-up of a PrEP implant targeted to at-risk populations, including adolescent girls (AG), young women (YW) under 30, and female sex workers (FSWs) in South Africa.

## Methods

### Study setting

We conducted qualitative in-depth interviews (IDIs) to understand PrEP implant perceptions amongst health care providers (HCPs) and end-users including adolescent girls (AG, ages 15–17), young women (YW, age < 30) and female sex workers (FSWs, age > 18) across rural/peri-urban and urban settings in Soshanguve township, Gauteng Province, South Africa.

### Study population

We recruited HCPs from participating public sector clinics and hospitals in the study areas. Healthcare workers were eligible to participate if they were working as nurses/nurse practitioners, pharmacists, or general practitioners (GPs) in the public sector at the time of recruitment. The sample was split between providers who had experience offering long-acting contraceptive products and/or oral PrEP, and those who did not.

We limited recruitment of FSWs to adult women 18 years of age and older who had exchanged sexual services for cash or payment in kind at least once within the previous 6 months. AG, YW, and FSWs were eligible to participate if they were residents of the study area at the time of recruitment. Participants were divided between users and non-users of long-acting implantable contraceptive devices and/or oral PrEP. Participants who were unwilling or unable to provide informed consent, or who were unwilling to be audio-recorded, were ineligible for the study.

### Participant recruitment

We approached leadership at public clinics, hospitals, and health facilities in Soshanguve township to recruit HCPs. Target end-users were also recruited from Soshanguve township. Neighborhoods were selected based on district HIV prevalence estimates derived from StatsSA data. AG and YW were recruited from high traffic areas (e.g., shopping malls), while FSWs were recruited from shebeens (unlicensed establishments selling alcohol) and taverns. A snowball sampling approach was utilized for FSW recruitment, whereby the initial primary respondent was approached by a gatekeeper who asked her to refer other interested FSWs to the study research team. Respondents were selected for inclusion in the IDIs to maximize diversity in terms of age, experience with oral PrEP and/or implantable contraceptive devices, and other demographic characteristics. With regards to data saturation, the research team used a simple-to-apply method applied to inductive thematic analysis [16].

### Data collection

**Fig. 1:**
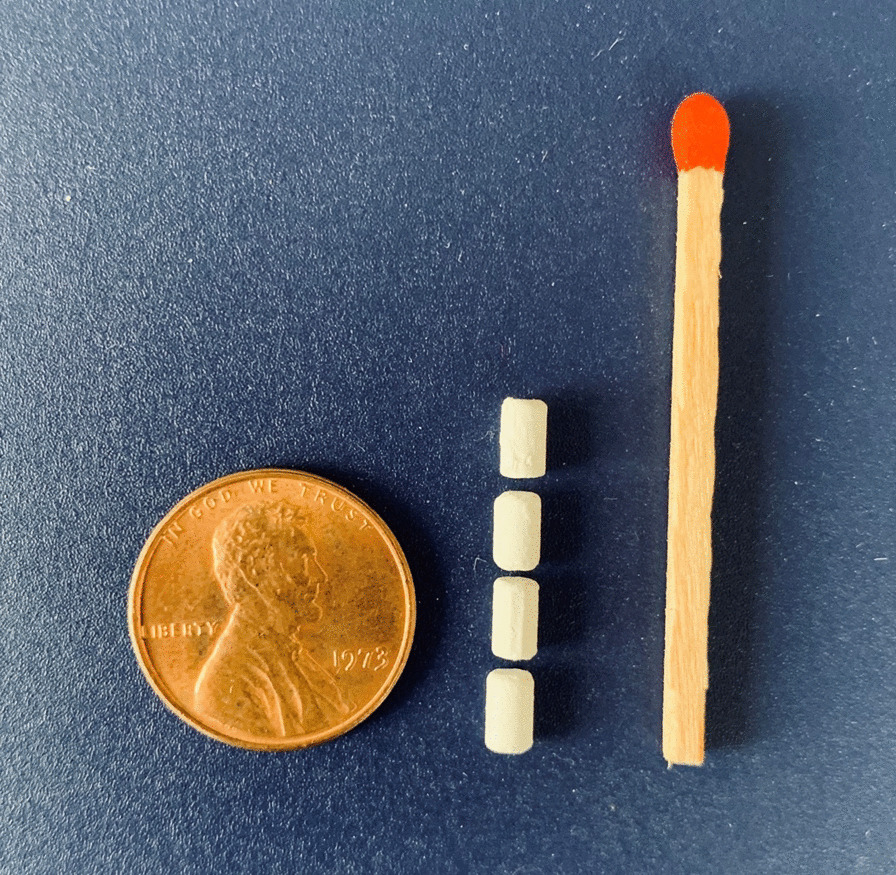
ARV-releasing pellet prototype, under development by CONRAD

Consenting participants completed a 60–90 min IDI using a semi-structured interview guide administered in private spaces by a trained researcher in the language of the participant’s choosing. Provider interviews focused on acceptance, willingness, barriers, and motivators to the prescription/provision of implantable PrEP. Target end-user interviews were focused on preferences, values, beliefs, habits, lifestyles, influencers, and information channels that influence implantable PrEP uptake. Visual aids and/or prototype examples were used to illustrate the design features of the implantable PrEP product and insertion device for both HCPs and end-users (see Fig. 1).

### Ethics statement

The study protocol was approved by the PSI Research Ethics Board and the University of the Witwatersrand Institutional Review Board. All respondents provided informed consent to participate. AGs between the ages of 15–17 provided informed assent and needed parental consent for participation. Parents and or guardians of AGs provided consent for their children to be interviewed in a private location with a trained interviewer. End-user participants received 100 ZAR (approximately USD $5.40) to compensate them for their time and travel.

### Data analysis

Interviews were recorded, transcribed, and translated into English. Transcripts were de-identified and analyzed in Dedoose using an inductive analysis approach to identify new themes as they emerged, and a deductive approach to explore themes pre-identified by the research team. Two investigators conducted thematic analyses and read the transcripts several times, noted preliminary themes, produced initial codes, reviewed and refined themes, and generated a final coding framework for analysis.

## Results

From May–June 2019, a total of 36 respondents were recruited to participate in IDIs (Table 1).

**Table 1:** Qualitative study sample of healthcare providers and target end-users

Primary respondents	Experience providing PrEP or implantable contraceptive	No experience providing PrEP or implantable contraceptive	Total
Nurse/Nurse Practitioner	2	2	4
Pharmacists	2	2	4
General Practitioner (GP)	2	2	4
Total	6	6	12

Secondary respondents	Urban*	Rural/Peri-urban*	
Adolescent girls (15–17)	4	4	8
Young women (18–30)	4	4	8
Female sex workers (18+)	4	4	8
Total	12	12	24

^*^Split between users and non-users of implantable contraceptives and/or oral PrEP

The sample included 12 HCPs, half of whom had experience providing implantable contraceptives or PrEP (n = 6), while the remaining half had no direct experience offering these services. Providers included nurses/nurse practitioners (n = 4), pharmacists (n = 4), and general practitioners (GPs, n = 4). The sample also included 24 target end-users, divided across AG (n = 8), YW (n = 8), and FSWs (n = 8) and urban (n = 12) and rural/peri-urban (n = 12) locations.

### Experiences with oral pre-exposure prophylaxis

Overall end-user awareness of oral PrEP was low: only 1 AG, 1 YW, and 1 FSW had heard of PrEP before the study. Despite low awareness, once the concept of oral PrEP was explained to them, most end-users reacted positively towards the product. End-users viewed oral PrEP as suitable for all sexually active women and girls, and of particular value to FSW who have increased risk of contracting HIV. However, end-users were concerned about oral PrEP use being misunderstood by others, resulting in rumors of their HIV status or promiscuity. AG especially worried about remembering to take the pill every day, and the consequent HIV risks.

“Yes, it is a challenge, what if I forget to take it, will I be safe? I think if I forget it for only a day I will still be protected? If you could take one tablet a month that will help because you do not need to remember every day.”—Adolescent Girl 1

While YW liked the idea of being protected from HIV, they were also concerned about the privacy of their PrEP use to avoid assumptions from others that they were HIV positive should they see them at the clinic or catch them in possession of pills. However, the security gained from a highly efficient HIV prevention product overrode many concerns about privacy.

“I’d use it because I don’t know what life has in store for me. You might meet a person that doesn’t look sick, you don’t know their results, or you get raped and you don’t go to the clinic on time. so, I would use it.” —Young Woman 1

Many FSWs liked the ability to be protected from HIV without requiring a customer to wear a condom. While adherence to oral PrEP was sometimes noted as a potential challenge, many thought the benefits of HIV protection would outweigh the burden of a pill each day and were confident that they could develop a routine to fulfil the daily dose requirement.

“I think PrEP would be right for me as a sex worker because if you know that you are not going to use protection or something happens even as you use protection, you know you are still protected.”—Female Sex Worker 1

Most providers (11/12) in our sample were aware of oral PrEP. HCPs, particularly GPs, noted that members of high-risk populations, such as serodiscordant couples, MSM, sex workers, and those with risky sexual behavior had already expressed interest in oral PrEP. The providers viewed PrEP as benefiting a wider range of people including everyone who is sexually active, from the ages of 14–55+.

“Eh, everyone who is in a high-risk relationship. Those would be, let’s say your normal sexual couples, where let’s say one of the partners is HIV positive ehm, you've got people who are seeking employment their bodies for example. Prostitutes, sex workers, they should be, yes that’s highly recommended.”—Healthcare Provider 1

However, many HCPs expected their clients to have challenges taking a daily pill, especially among younger people, who often forget or dislike taking pills. Providers also expressed concerns about the affordability of PrEP, its potential side effects, the limited availability in public health facilities, and the lag in HIV protection after initiation. Providers also told us that oral PrEP is often seen as being treatment for HIV infection, rather than prevention, given that the pills are the same as those used for HIV treatment. This could lead to stigma and discrimination for end-users, who may discontinue PrEP as a result. Finally, HCPs, especially pharmacists, also feared that PrEP scale-up would lead to declines in condom use, normalizing risky sexual behavior and putting PrEP users at risk of unintended pregnancy or other sexually transmitted infections (STIs).

### Reactions to the PrEP implant

Reactions to a biodegradable PrEP implant were generally positive among AG, YW, and FSWs. With regards to the PrEP implant, respondents mentioned increased happiness and safety at the prospect of being protected against HIV.

“I am happy because It’s protecting my life, so I don’t care even if it’s visible.”*—*Female Sex Worker 2

“This one you are safe all the way with the implant.”—Young Woman 1

Though some potential aspects of the implant (e.g., side effects, pain during insertion, scarring) were seen as undesirable, most respondents felt that these would be offset by a highly effective HIV prevention product. As one FSW noted: “I can withstand any pain as long as my life will be ok”.

“I would rather endure 2 min of pain than to contract HIV. If I could survive labor pains, then that little cut won’t kill me.”—Female Sex Worker 2

Women noted that side effects from the PrEP implant would be “bearable” or able to be “tolerated” as long as: (1) the product provided a high degree of protection against HIV (typically defined as at least 90% protection), (2) women were counselled on potential side effects before insertion and knew what to expect in terms of symptoms and their likely duration, and (3) women were able to create a plan for dealing with side effects with their provider in advance of the insertion.

“I think before they insert it, they have to tell me more about it and its side effects so that I know what to expect once it has been inserted and not find after insertion I am throwing up and want to take it out.”—Adolescent Girl 3

Women viewed the product’s ability to dissolve as a benefit, primarily because it would cut down on facility visits and allow women to avoid a potentially painful removal process. However, women and HCPs were both concerned that end-users would be stuck—potentially for long periods of time—with a non-removable device that was no longer wanted due to side effects. While a longer duration of protection (a year or more) was generally desired, this was in tension with the idea that the product could not be removed.

“What if it doesn’t treat you well for the first 2 weeks and you want you have it removed and you have a continuous headache, then you must wait for the 6 months or the 3 months?”—Young Woman 1

Women and providers both suggested the possibility of “trial dose” consisting of a shorter-lasting PrEP implant (e.g., 1–3 months) so that women could see if they liked the product and could manage the side effects before committing to the full dose.

Many respondents said they would want to share their experiences with PrEP to benefit family, close friends, and even partners.

“I would tell my friend that there is something at the clinic for prevention against HIV and it’s not difficult. We just go to the clinic and they check us out for all our sicknesses and if you are all right then then they inject this thing into you and this thing dissolves inside your body and you will not feel anything and that we should go and try it.”—Young Woman 3

The discreet delivery of PrEP products within general health or even FP clinic spaces (as opposed to HIV spaces) was recommended by several of our respondents to make uptake less stigmatizing.

“if I sit in the HIV line people will speculate that I’m also HIV positive. So, people will assume I am there for family planning and it will be just that. So, I would choose family planning side. Not the HIV side. ” —Young Woman 2

End-users expressed a high degree of willingness to try a PrEP implant if it were available, and felt that their peers would be likely to try it as well. One FSW said, “I would use it as it will help me in my line of work.” Another said “I would use the product because it means for my work I don’t have to worry about those clients who don’t want to use a condom.” Most YW liked the fact that the implant would protect from HIV without taking a daily pill and liked the convenience of a single, dissolvable insertion, which meant they wouldn’t have to visit the clinic for a removal. Similarly, AG were largely positive about the PrEP implant concept.

“I love the idea because many South Africans are HIV positive, so those that are still negative will be protected from HIV and the death rate will decrease.”—Adolescent Girl 4

“People are aware of the existence of HIV so to have this information that now there’s something that can protect you, number one that will be appealing to the people, to the youth, number two the most eh, the other thing that will be appealing is this thing that will protect you is something that you put once and it’s there because people don’t want to take pills…it will be very appealing that there’s this device that can be inserted once off and repeated a long time later you see, it will be very appealing.” Healthcare Provider 2

HCPs recognize the discreeteness value-add attribute of a PrEP implant. Several providers commented on how the one-insertion product will remove adherence barriers for clients who don’t desire, or can't remember to take a daily pill. A few providers claimed that the product would be appealing to their young clients.

While providers were mostly positive about the potential for the PrEP implant, as with oral PrEP some providers feared that the availability of implantable PrEP would lead to increases in promiscuity and risky sexual behavior, and ultimately to an increase in STIs, pregnancy, or rape. These providers, who tended to be pharmacists, expressed preferences for post-exposure prophylaxis (PEP), which they thought could be used for “any unforeseen possible HIV exposure”. Some HCPs were concerned about possible implant side effects, or felt that the implant’s inability to protect against all STIs was a shortcoming.

Despite these misgivings HCPs saw two key benefits from implantable PrEP: (1) the long duration of protection afforded by the product; and (2) the likelihood of higher adherence relative to oral PrEP, given it would not be a user-driven pill. Providers liked the dissolvability of the device, noting this feature would benefit both patients, who would not have to undergo the painful device removal process, and relieve workload from healthcare facilities and providers who would otherwise have to perform removals. In addition to these benefits, HCPs also liked the fact that implantable PrEP is discreet and requires little to no maintenance from the user. These benefits were seen as improvements on oral PrEP, which can be difficult for patients to keep a secret from others.

## Discussion

While oral PrEP awareness and experience was generally low in our end-user sample, AG and YW were eager to have options to protect themselves from HIV. All of our respondents expressed concern about their risk for HIV, including having multiple sexual partners (or partners who likely themselves had additional sexual partners), frequent instances of unprotected sex, and experiences of sexual assault. These findings are consistent with other studies which have identified multiple levels of HIV risks faced by adolescent girls and young women in South Africa [17]. Women and girls repeatedly used the words “protection” and “safety” when describing PrEP, words which evoke feelings of increased security, control, empowerment and ultimately, the desire for peace of mind in an HIV prevention product. Previous research on oral PrEP has also found high levels of self-reported acceptability, including among high-risk women in South Africa [18–21].

Despite this enthusiasm, the AG and YW in our study expressed reservations about oral PrEP. These concerns fell into two broad categories: (1) Fear of forgetting to take a daily pill, and (2) Negative community assumptions about PrEP users. AG and YW expressed concerns that anyone seeing them taking the pills would assume that they were either promiscuous or already living with HIV. These findings are largely in line with previous research that has identified stigma as a barrier affecting uptake of and adherence to oral PrEP [19, 21–24]. By being more discreet and obviating the need for daily user action, the PrEP implant could improve acceptability of PrEP and address the target population’s two primary adherance concerns.

Overall, women in our study said they were willing to tolerate at least some implant side effects (especially if they were short-lived) but wanted to be fully counseled on what to expect, including the types, likelihood, and duration of potential side effects. They also wanted to have plans in place to deal with any symptoms (e.g., analgesics for headaches) prior to insertion. This sentiment is shared amongst end-user experiences with analogous products such as the contraceptive implant or oral PrEP [25, 26].

Women and girls appeared willing to make substantial trade-offs in terms of insertion pain and side-effects if it meant they could have peace of mind with an effective HIV prevention product. However, women’s expectations of what constitutes an effective PrEP implant were high. Most thought that an implant should be at least 90% effective at preventing HIV if they were to use it. While substantially lower levels of effectiveness might generate substantial population-level reductions in HIV incidence, this level of protection may not be considered sufficient for many potential end-users. Careful framing of product effectiveness will likely be important for uptake, especially if a product is unable to achieve such high levels of protection against HIV. Additional research is needed to determine what product features end-users might be willing to forego (e.g., shorter duration of protection) or tolerate (e.g., greater side effects) for a relatively more effective product.

Despite a desire for a long-acting product, multiple respondents suggested a “trial dose” (e.g., a smaller dose with a shorter duration of protection) prior to the full implant being inserted so that they could see how their bodies responded to the product. The CONRAD implant is designed to have a window of removability should adverse effects arise shortly after implantation; however, respondents feel that providing a “trial dose” could assuage fears that accompany trying a new product. Further, giving women this option may increase uptake by reducing the number of attempted removals and negative experiences with the product. Mimizing negative experiences with the product is especially important to curbe any adverse information that may be shared to other potential end-users, which could potentially dampen enthusiasm for the product among peer groups. An oral trial may be possible, depending on availability of the drug in oral forms, and has been used in Phase III clinical trials [27, 28]. A short-term implant may be another option, though this would potentially require additional insertions/removals depending on whether a full “dose” was desired after the trial. In a follow up discrete choice experiment, we plan to investigate the option for biodegradable implant that could be removed during a short period of time, e.g., 1 month, in case safety issues arose or they changed their minds [27, 28].

Providers are also likely to play an important role in implantable PrEP demand. Nurses and doctors are trusted arbiters of information about reproductive health [29, 30], and those involved in family planning specifically will likely have an important role to play in increasing awareness of the PrEP implant. Providers will play a key role in ensuring women and girls have their questions answered prior to insertion, have the right expectations, and are well-prepared to deal with any potential side effects. Should AG and YW have accurate expectations of side effects and insertion pain, they can develop potential strategies to deal with these eventualities and share their management plans with other potential PrEP implant users.

Provision of the implant within family planning spaces may also afford women and girls more privacy in accessing the product. FP providers are well-versed in discussing and providing long-acting contraceptives, which have numerous analogues to the PrEP implant, and these experiences should be leveraged to promote and provide this product once it is available. Despite some reservations about the product, providers were more willing than end-users to tolerate a lower level of implant efficacy. Relying on provider expertise, knowledge, and credibility to educate patients about acceptable efficacy levels and necessary additional protections will be critical to a successful uptake and maximum effectiveness.

### Limitations

Limitations of this study include a small sample size, and its exclusive focus on high-risk female populations. While the current research focus is on developing next-generation HIV prevention options for women, the PrEP implant could certainly be considered and tested amongst high-risk male populations. Moreover, participants were mostly recruited in urban/peri-urban areas and from a single province in South Africa. Lastly, awareness of and experience with oral PrEP among end-user participants was low, therefore, most respondents did not have direct experiences with PrEP products prior to this study.

## Conclusion

Our research indicates a strong desire for more convenient, discreet, and effective biomedical HIV prevention option, afforded by a PrEP implant, among at-risk women in South Africa. While ongoing related research is evaluating the specific product features that might drive uptake of a PrEP implant, and the relative magnitude of these preferences, this work suggests a strong demand for a long-acting, implantable, and preferably biodegradable PrEP product. Given our small sample size, futher research, including mixed method studies should be conducted to produce more generazible results for end-users.

## Data Availability

The qualitative data (interview transcripts) used and/or analyzed during the current study are not publicly available because they could not be fully de-identified. Qualitative interview guides and codebooks are available, along with redacted versions of the transcripts, from the corresponding author on reasonable request.
